# An Atopic Dermatitis-Like Mouse Model by Alternate Epicutaneous Application of Dinitrofluorobenzene and an Extract of Dermatophagoides Farinae

**DOI:** 10.3389/fmed.2022.843230

**Published:** 2022-06-15

**Authors:** Shujing Feng, Wengying Liu, Sisi Deng, Guoxuan Song, Jie Zhou, Zhengni Zheng, Zhiqiang Song

**Affiliations:** ^1^Department of Dermatology, Southwest Hospital, Army Medical University, Chongqing, China; ^2^Changsha Medical College, Changsha, China

**Keywords:** atopic dermatitis, BALB/c mice, house dust mite, Dermatophagoides farina, dinitrofluorobenzene

## Abstract

Several studies have tried to establish mice models of atopic dermatitis (AD) through the allergen of *Dermatophagoides farinae* (Df). However, there are no typical skin lesions after epicutaneous application of an extract of Df (DfE) on BALB/c mice. Dinitrofluorobenzene (DNFB) is a common hapten that brings about contact dermatitis. Skin dysfunction induced by DNFB may be a way to enhance the effects of DfE on mice skin. Thus, we hypothesized that alternate epicutaneous application of DNFB and DfE could induce AD-like skin lesions on BALB/c mice. To test this hypothesis, we alternately applied the DNFB and DfE to the back skin of BALB/c mice for 8 weeks. Changes in mice skin lesions and the frequency of scratching behavior were recorded. The variation of Th1-related cytokines (interferon-γ [IFN-γ] and interleukin two [IL-2]) and Th2-related cytokines (IL-4 and IL-13) was detected in serum and lesional skin. Eventually, the BALB/c mice developed severe erythema, erosion, scarring, and excoriation on the entire back, showing a high frequency of scratching behavior. In addition, Th2 cells' dominant cytokines appeared in both serum and lesional skin. Those results indicate that alternating epicutaneous exposure to DNFB and DfE can produce AD-like models with typical clinical features and Th2-type immune responses in BALB/c mice. This model could be valuable for studying the pathogenesis of AD and developing novel therapeutic agents for it.

## Introduction

Atopic dermatitis (AD) is a chronic relapsing inflammatory skin disease that affects about 20% of children and 10% of adults in developed countries (1). Patients with AD often have a family history of other allergic diseases, such as allergic rhinitis, rhinoconjunctivitis, and asthma (2). Apart from intense pruritus and dryness of the skin, the clinical features of AD are usually highly heterogeneous and vary by age and region (3). Abnormal skin inflammation plays a key role in the pathological development of AD, and it is usually triggered by skin barrier dysfunction and microbial imbalance under interactions between genetic and environmental factors (4, 5). For this abnormal inflammation, Th2-type immune responses, the pivotal feature of AD, predominate over other types of inflammation and produce cytokines such as interleukin (IL)-13 and IL-4, especially in the acute phase (6). In addition, IL-13 is preferentially involved in peripheral tissues because tissue-resident innate lymphoid cells produce IL-13 but not IL-4 (7). Notably, the Th2-type immune responses are not only the local production of Th2-type cytokines but also prolonged survival and activation of eosinophils, mast cells, and production of serum allergen-specific IgE (8).

In recent decades, a variety of animal models of AD have appeared, which have received increasing attention (9, 10). It is essential to create an animal model similar to patients with AD as much as possible so that people can use this animal platform to deepen their understanding of the pathogenesis of AD and conduct novel therapeutic drug research. On account of cutaneous allergen sensitization, a critical and early event in the pathogenesis of AD, several AD animal models have been built by the epicutaneous application of exogenous allergen (11–14). House dust mites are the most important allergen sources worldwide, and the majority of atopic patients have high titers of specific IgE to it (15, 16). Further studies have demonstrated that house dust mites, especially *Dermatophagoides farinae* (Df), play a major role in the pathogenesis of AD, promoting the Th-2 type immune responses and impairing the function of the skin barrier (17). Using the topical application of a crude extract of Df (DfE), Matsuoka et al. (12) successfully established an AD mouse model in NC/Nga mice, which showed high levels of specific IgE in the sera and AD-like skin lesions only in the head and neck areas (12). Interestingly, in their report, when BALB/c mice were stimulated with DfE, serum specific IgE increased but no skin lesions appeared.

It is meaningful and necessary to use DfE to establish an AD-like mouse model in common BALB/c mice, which exhibit not only typical skin lesions but also Th2-type immune responses but were faced with the problem of triggering AD-like skin lesions. Dinitrofluorobenzene (DNFB) is a common hapten to induce contact dermatitis in mice (18, 19). The integrity of mice skin gets disrupted when DNFB recruits the cytotoxic T lymphocytes to the skin, inducing keratinocyte apoptosis (20, 21). The skin dysfunction caused by DNFB may be a way to enhance the effects of DfE on mice skin. Thus, we hypothesized that the alternate epicutaneous application of DNFB and DfE could induce AD-like skin lesions and Th2-type immune responses in BALB/c mice. To test this hypothesis, we alternately applied the DNFB and DfE to the back skin of BALB/c mice for 8 weeks. Changes in mice skin lesions and the frequency of scratching behavior were recorded. The levels of DNFB and DfE specific IgE in serum were detected. Furthermore, the variation of Th2-related cytokines (IL-4 and IL-13) and Th1-related cytokines (interferon (IFN)-γ and IL-2) was detected in serum and lesional skin. To better understand this model, we also scrutinized the characteristics of the BALB/c mice with the repeated application of DNFB or DfE.

## Materials and Methods

### Animal and Sensitization

Specific pathogen-free, 6-weeks-old female BALB/c mice were purchased from the Experimental Animal Center of the Army Military Medical University (Third Military Medical University, Chongqing, China). Mice were housed in a carefully controlled environment, with a steady temperature (22 ± 1°C), humidity (55 ± 10%), and a 12-h light-dark cycle. Then, 1 weeks after arriving, the hairs of the entire back were removed from anesthetized mice using an electric shaver and depilatory protocol. Hair removal was performed every 1–2 weeks. All experiments were approved by the Laboratory Animal Welfare and Ethics Committee of Third Military Medical University and followed the National Institutes of Health Guide for the Care and Use of Laboratory Animals.

**Figure 1:**
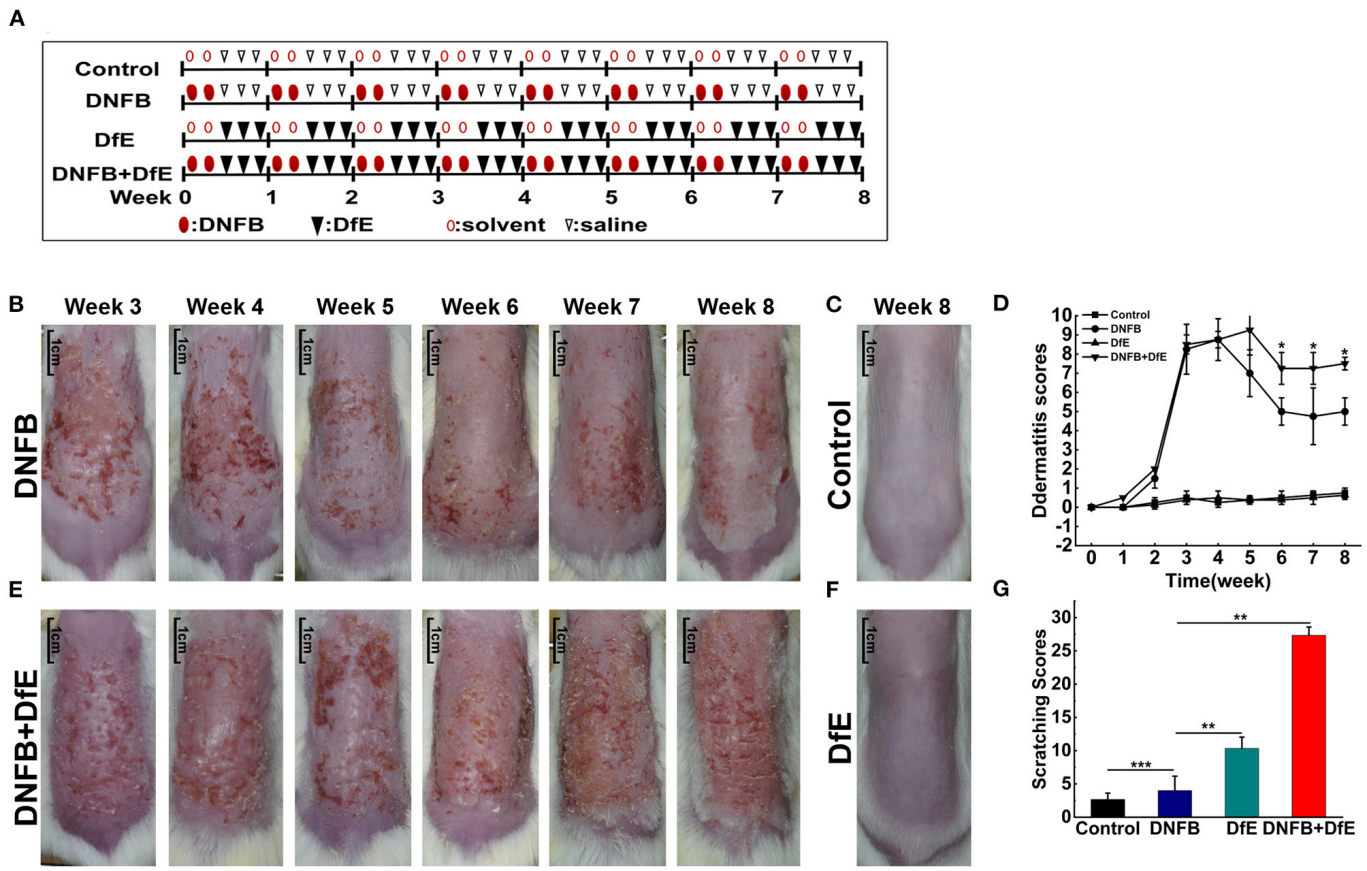
Atopic dermatitis (AD)-like clinical features were induced by alternate application of dinitrofluorobenzene (DNFB) and an extract of Dermatophagoides farinae (DfE) in BALB/c mice. **(A)** Experimental design. BALB/c mice were randomly divided into 4 groups (*n* = 4), including the control group, the DNFB group (100 μL of 0.15% DNFB, two times a weeks), the DfE group (100 mg DfE ointment, three times a weeks), and the DNFB+DfE group (alternate application of DNFB and DfE). The mice of the control group were alternately exposed to the solvent (a 3:1 mixture of acetone and olive oil) and saline. All reagents were repeatedly applied to the dorsal hairless skin for 8 weeks; **(B–E)** Skin lesions in the DNFB group, the control group, the DNFB+DfE group, and the DfE group. (Scale bar = 1 cm); **(F)** Changes of dermatitis severity. Dermatitis scores were based on the symptoms of erythema, edema, dryness/scarring, and excoriation/erosion, and were compared between the DNFB+DfE group and the DNFB group (*n* = 4 mice); **(G)** Scratching behavior after 7 weeks of treatment. The scratching behavior was expressed as points (as shown in Materials and methods) accumulated for 10 min. Scratching scores are representative of three mice in each group. Data are showed as mean ± standard deviation (SD). *p < 0.05, **p < 0.01, and ***p < 0.001.

After hair removal, BALB/c mice were randomly divided into four groups (*n* = 4/group), including the control group, the DNFB group (100 μl of 0.15% DNFB dissolved in a 3:1 mixture of acetone and olive oil, two times a weeks), the DfE group (100 mg DfE ointment, three times a weeks), and the DNFB + DfE group (alternate treatment with DNFB and DfE). The control group alternately used the solvent (a 3:1 mixture of acetone and olive oil) and saline. All reagents were softly and evenly applied to the dorsal hairless skin for 8 weeks. DNFB was obtained from Xiya reagent, Shandong, China, and DfE ointment were provided by Biostir-AD, Biostir Inc., Kobe, Japan (Figure 1A).

### Evaluation of Dermatitis Severity

The severity of dermatitis was measured based on symptoms of erythema, edema, dryness/scarring, and excoriation/erosion. Each symptom was scored on a scale of zero (none), one (mild), two (moderate), or three (severe). Total dermatitis scores were the sum of these individual scores. At the end of each weeks treatment, each symptom score was assessed by two independent researchers. Photographs were obtained using identical camera settings and lighting each weeks.

### Scratching Behavior

Apart from daily observations, scratching behavior got recorded the next day morning after 7 weeks of treatment. The scoring rule was a modulation of previous standards to fit our mice models (12). Briefly, scoring was performed at 1 min intervals by a volunteer who was unaware of the treatment status. Points were scored as follows: (1) if the mouse scratched once; (2) if it scratched for few seconds; and (3) if it scratched for 10–15 s, 4 if it scratched for 15–20 s, and 5 if it scratched for more than 20 s. Scores were accumulated from 0 to 10 min before the application of the extract.

### Histological Analysis

Dorsal skin tissues were obtained from the same site and fixed in 10% neutral formalin and embedded in white paraffin. Serial paraffin-sections of 4 μm thickness, were prepared and stained with hematoxylin-eosin (H&E) or toluidine blue. The tissue slides were converted into digital images and analyzed through the iviewer image analysis software (UNIC Technologies, Beijing, China). Mast cells, stained by toluidine blue, and eosinophils by H&E were counted in three different fields of 0.250 × 0.250 μm and averaged.

### Measurement of Serum IgE by ELISA

The next day, after the final application (12th) of the extracts, mice were killed and blood was taken to collect sera stored at −80°C. Serum levels of total IgE, DfE specific IgE (DfE-sIgE), and DNFB specific IgE (DNP-sIgE) were measured using ELISAs (j&l biological, Shanghai, China) according to the instructions of the manufacturers.

### Serum Protein Quantitation by Multiplex Assay

Samples were thawed and proteins (IL-2, IL-4, IL-13, and IFN-γ) were quantified using a LEGENDplexTM Mouse B Cell Panel immunoassay (BioLegend, United States) according to the manufacturer's instructions. Data were acquired from the Gallios Flow Cytometer (Beckman Coulter, United States) and analyzed using software provided by BioLegend.

### Real-Time PCR Analysis

Total RNA was isolated from skin samples using RNAiso Plus (TaKaRa, Dalian, China) according to the instructions of the manufacturer. The cDNAs were synthesized using the PrimeScriptTM RT reagent Kit (TaKaRa). Real-time PCR was performed with TB GerrnTM Premix Ex TaqTM II (TaKaRa) on a CFX ConnectTM Real-Time System (BIO-RAD, Singapore, United States). The reaction parameters were as follows: 95°C for 30 s, then 40 cycles of 95°C for 5 s, and 55°C for 45 s. The expression levels were calibrated to the β-actin control and determined by the 2-ΔΔCt method. The primers used were as follows: IL-2 forward, 5'-TGTGGTGGACTTTCTGAGG-3' and reverse, 5'-AGGGCTTGTTGAGATGATG-3'; IL-4 forward, 5'-GCACGGAGATGGATGTG-3' and reverse, 5'-CAAGCATGGAGTTTTCCC-3'; IL-13 forward, 5'-GCCAGCCCACAGTTCTAC-3' and reverse, 5'-AGACCACCAAGGCAAGC-3'; IFN-γ forward, 5'-TGAGGTCAACAACCCACA-3' and reverse, 5'-ACTCCTTTTCCGCTTCCT-3'; β-actin forward, 5'-CTCTTCCAGCCTTCCTTCCT-3' and reverse, 5'-AGCACTGTGTTGGCGTACAG-3' (Sangon Biotech, Shanghai, China).

### Statistical Analysis

The results were expressed as mean ± standard deviation (SD). All statistical analyses were performed using SPSS version 23.0 (IBM, Armonk, New York, United States). All data that followed a normal distribution were tested with a two-tailed Student's *t*-test or one-way ANOVA. Statistical significance was defined as *p* < 0.05. Significance levels of data were denoted as ^*^ *p* < 0.05, ^**^ *p* < 0.01, and ^***^ *p* < 0.001.

## Results

### Typical Skin Lesions Following Alternate Exposure to DNFB and DfE

To compare the effects of different exogenous agents on the skin of mice, changes in the skin lesions were recorded (Figures 1B–F). After a 3 weeks induction, the BALB/c mice developed severe erythema, erosion, scarring, and excoriation on the entire dorsal skin in the DNFB group and DNFB+DfE group. However, with relief of clinical symptoms and decline of dermatitis scores to a plateau over time, the skin lesions were impressively more severe in the DNFB+DfE group. In addition, chronic skin lesions, characterized by scaly patches and plaques with excoriation and lichenification, were only seen in the DNFB+DfE group. The skin lesions did not occur in the DfE group and control group. The BALB/c appears AD-like skin lesions only after alternate exposure to DNFB and DfE.

### High Frequency of Scratching Behavior Triggered by Alternate Stimulation of DNFB and DfE

After 7 weeks of stimulation, we recorded the scratching behavior for 10 min and got a cumulative score (Figure 1G). The frequency of scratching behavior in the DNFB+DfE group and DfE group was significantly higher than in other groups, and the former was higher, although no statistical differences between them. The scratching behavior was also observed in the DNFB group and the control group, with very low frequency, and the former is higher. Those results show that the scratching behavior is mainly induced by DfE and aggravated by DNFB.

### Progressive Skin Dermatitis Induced by Alternate Exposure to DNFB and DfE

**Figure 2:**
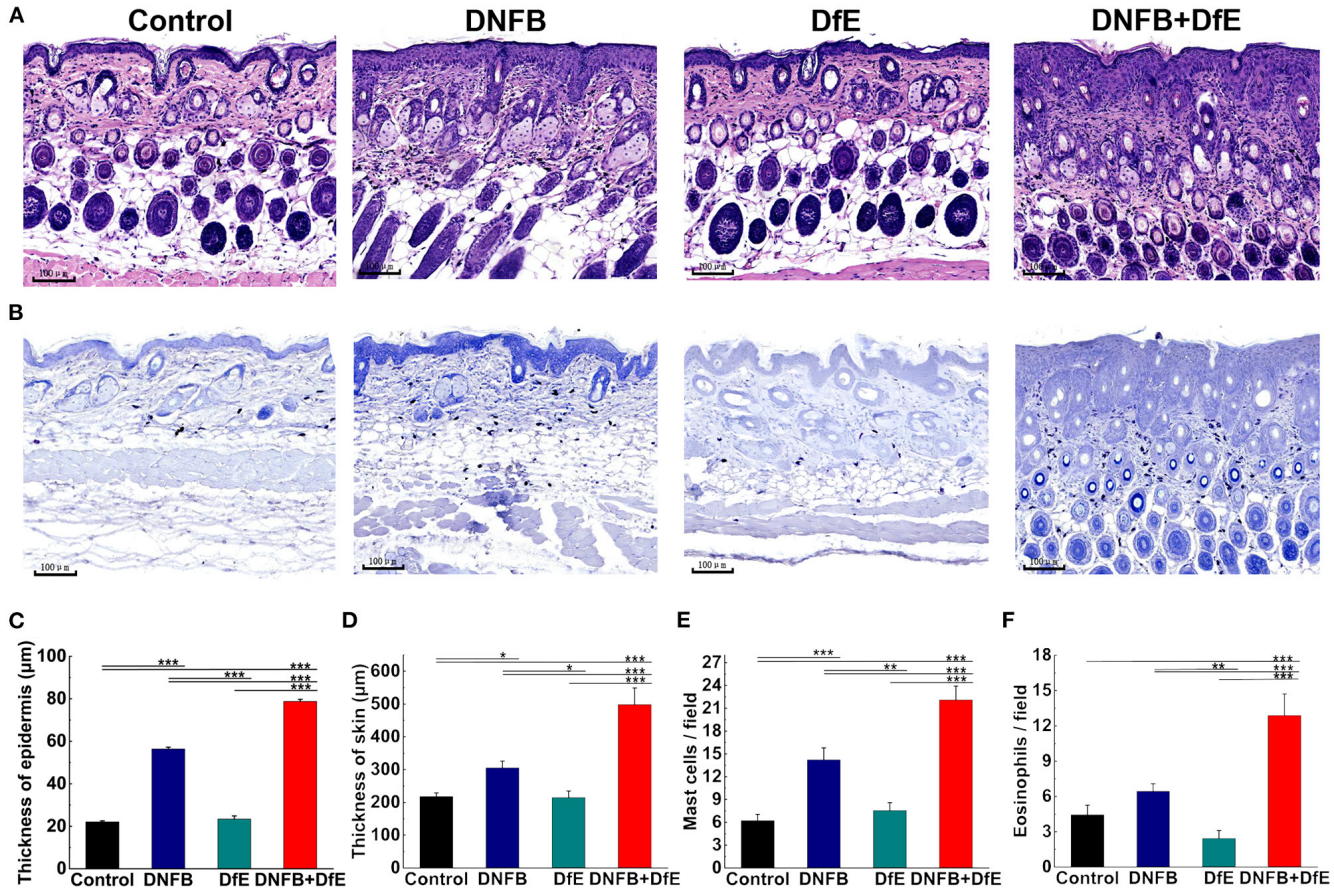
Skin dermatitis got largely promoted after 8 weeks of alternate application of DNFB and DfE. **(A)** Hematoxylin and eosin (H&E) staining; **(B)** Toluidine blue staining. (Scale bar = 100 μm); **(C)** Epidermal thickness; **(D)** Thickness of skin, including the epidermis and the infiltration depth of inflammatory cells in the dermis; **(E)** The average number of mast cells per field of 0.250 × 0.250 μm; **(F)** The average number of eosinophils per field of 0.250 × 0.250 μm. The number of mast cells and eosinophils is representative of three mice in each group (*n* = 4 mice). All data were measured using the iviewer image analysis software. Data are shown as mean ± SD. * *p* < 0.05, ** *p* < 0.01, and *** *p* < 0.001.

To further comprehend the alteration under the skin lesions, the histologic status was examined after 8 weeks of treatment (Figure 2). The thickness of the epidermis in the DNFB+DfE group and the DNFB group was thicker, and the former was more evident. There is no noteworthy difference between the DfE and the control mice. The thickness of skin, including the epidermis and the infiltration depth of inflammatory cells in the dermis, density of mast cells in the upper dermis, and density of eosinophils shared the same regularity as the thickness of the epidermis. Those results indicate that DfE aggravates the inflammation induced by DNFB.

### The Elevation of Serum Specific IgE to DNFB or DfE

**Figure 3:**
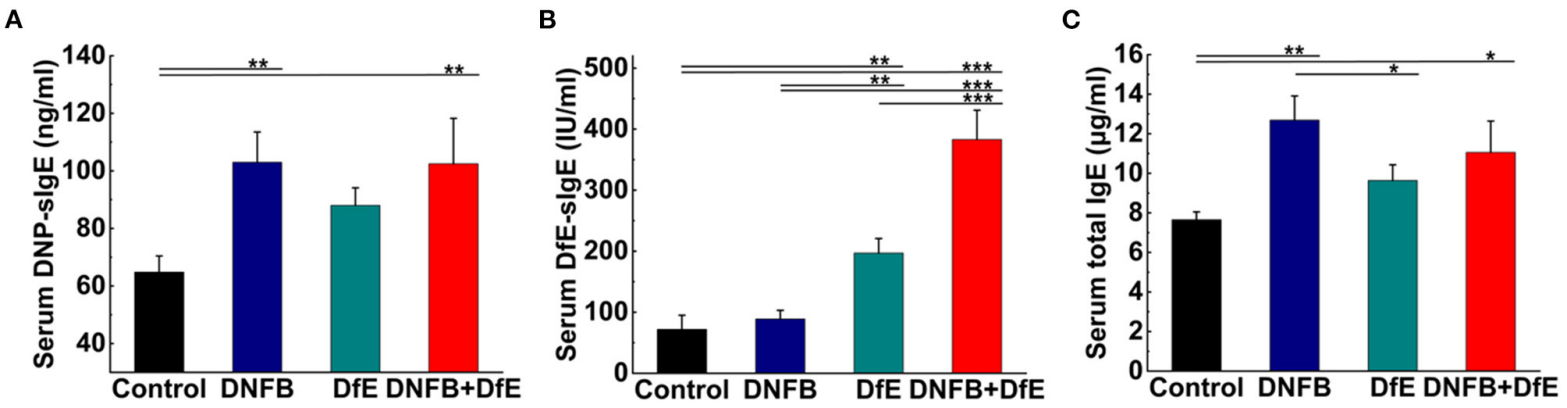
Serum specific IgE and total IgE increased after treatment with DNFB or DfE. ELISA analyses of serum. **(A)** DNFB specific IgE (DNP-sIgE); **(B)** DfE-specific IgE; and **(C)** total IgE. Results are representative of three mice in each group (*n* = 4 mice). Data are shown as mean ± SD. * *p* < 0.05, ** *p* < 0.01, and *** *p* < 0.001.

Exposed to the complex environment in daily life, patients with AD may exhibit several specific IgE antibodies against different exogenous antigens. In our study, the levels of serum DNP-sIgE and DfE-sIgE were increased after repeated application of DNFB or DfE on the dorsal skin of BALB/c mice. Moreover, the serum total IgE was improved in the DNFB group and the DNFB+DfE group, whereas no conspicuous differences between them. The single application of DfE just induced trivial elevation of the serum total IgE in BALB/c mice (Figure 3).

### The Discrepant Expression of a Serum Th1- and Th2-Related Cytokines

**Figure 4:**
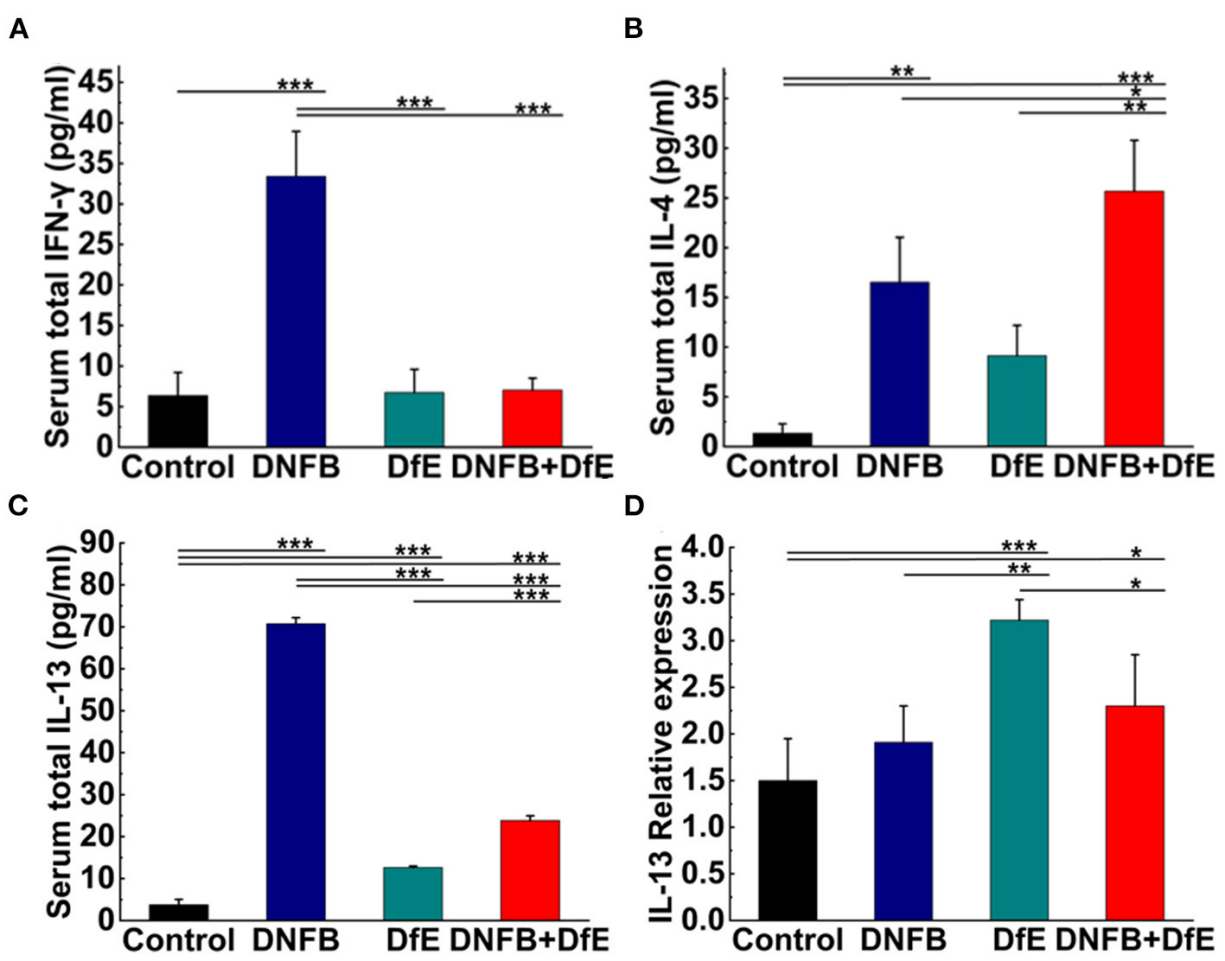
High levels of Th2-related cytokines were triggered by alternate application of DNFB and DfE not only in serum but also in lesional skin. Serum proteins of Th1-related cytokines. **(A)** interferon (IFN)-γ and Th2-related cytokines; **(B)** interleukin (IL)-4; and **(C)** IL-13. High levels of IL-2 appeared only in the DNFB group, but undetectable in other groups (data not shown). Results are representative of three mice in each group (*n* = 4 mice). Real-time PCR analysis of **(D)** IL-13 in mouse lesional skin. The expression of IL-13 mRNA is representative of four mice in each group (*n* = 4 mice). The expression of IL-4, IFN-γ, and IL-2 mRNA did not increase in the lesional skin of each group (data not show). Data are shown as mean ± SD. * *p* < 0.05, ** *p* < 0.01, and *** *p* < 0.001.

To study the dynamic changes of the cytokine milieu in serum, we used a multiplex assay panel to detect the production of cytokines proteins in the serum of BALB/c (Figures 4A–C). There were noteworthy high levels of Th2-type cytokines in the DNFB+DfE group, but not Th1-type cytokines. However, after repeated exposure to DNFB, the mice's serum Th1- and Th2-type cytokines were higher than any other group, except for IL-4 lower than the DNFB+DfE group. The mice serum level of total IL-13 also increased in the DfE group compared with the control group. These results show that Th2 cells predominate over the blood inflammation of mice in the DNFB+DfE group, while both Th1 and Th2 cells play a critical role in the blood inflammation of mice in the DNFB group.

### Th2-Related Cytokines Induced by DfE in Lesional Skin

The hallmark of AD is cutaneous inflammation, which is characterized by consecutive and progressive inflammatory cell infiltration, particularly by Th2 cells. Increased expression of IL-13 mRNA was detected in the lesional skin of mice in the DfE group and the DNFB+DfE group and the former is significantly higher, but not in mice in the DNFB group and the control group (Figure 4D). Nevertheless, the expression of mRNA for IL-4, IFN-γ, or IL-2 was not detected in all groups (data not shown).

## Discussion

Atopic dermatitis gradually becomes a burdensome disorder for human beings, owing to its symptoms of dry and itchy skin, sleep disturbances, anxiety, and depression (22). To study it deeply and conveniently, people pay close attention to animal models (10). Over the years, researchers have attempted to establish an ideal mouse model of AD through exogenous allergens of Df, but the skin lesions are not typical. In this study, due to the skin dysfunction caused by DNFB, we successfully produced an AD-like mouse model with not only typical clinical features but also Th2-type immune responses by alternately applying DNFB and DfE to the back skin of BALB/c mice.

During the 8 weeks of stimulation of DNFB and DfE, the dorsal skin of BALB/c mice got dry in the second weeks and skin erosion occurred in the third weeks. The typical and steady AD-like lesions appeared on the entire dorsal skin from 6 to 8 weeks. In addition, the mice in the DNFB+DfE group exhibited a high frequency of scratching behavior, exacerbating the dorsal skin lesions in the last few weeks. At the same time, the thickness of the epidermis and the infiltration of inflammatory cells, especially mast cells in the upper dermis, increased remarkably. What‘s more, the levels of serum total IgE, DfE-sIgE, and DNP-sIgE were increased in BALB/c mice. On the other hand, after repeated exposure to DNFB, the back skin of mice was mildly damaged, and some erythma and erosion appeared after the acute skin injury in the 3rd and 4th weeks. The thickness of the epidermis and the infiltration of inflammatory cells increased to some extent. High levels of serum total IgE and DNP-IgE were also induced by DNFB. Furthermore, when treated with DfE, the mice's dorsal skin did not show evident lesions and infiltration of inflammatory cells, but their serum DfE-sIgE was increased, and the serum total IgE was slightly increased as well. Those results indicate that the DfE, as a protein antigen, can give rise to an IgE-associated immune response, and the DNFB, as a chemical hapten, can bring about contact dermatitis. Above all, alternating exposure to DfE and DNFB can lead to typical AD-like skin lesions, intense itching, and scratching in BALB/c mice.

Cutaneous inflammation, characterized by Th2 cell polarization in acute skin lesions, is the center of the pathogenesis of atopic dermatitis (6, 8). In the DNFB+DfE group of our study, the dorsal lesional skin expressed a high level of IL-13 mRNA and the serum proteins of IL-4 and IL-13 increased as well. Those results further confirmed the success of our AD-like mice model.

In our study, DNFB enhanced the effects of DfE on BALB/c mice, resulting in more severe skin dermatitis and a higher frequency of scratching behavior. However, the mechanism by which DNFB affects the effects of DfE remains unclear. DNFB-induced contact dermatitis may impair the skin barrier, and skin dendritic cells are more likely to capture external protein antigens through the damaged skin barrier (23, 24). Immune modification by DNFB may be involved in this process (25). Besides, other factors also have an important influence on the stimulating ability of house dust mite antigens, such as antigen purity, dosage, and duration of action (13, 14, 26). In daily life, patients with AD are exposed to a complex environment of various haptens and protein antigens, and it may be necessary and meaningful to further study the mechanism of their interactions.

In the DNFB group, there were high levels of Th1- and Th2-type cytokines in the serum, but their mRNA expression could not be detected in the dorsal skin. In contrast to our study, a previous trial showed that high levels of IFN-γ and IL-2 mRNA expression were detected in the ear of mice, following repeated exposure to DNFB (19). However, unlike the previous trial, we applied the DNFB on the dorsal skin instead of the ear, and we obtained the specimens of lesional skin a few days, rather than hours, after the application of DNFB. Those differences may account for the different results in the DNFB group.

In the DfE group, the secretion of IL-13 proteins in serum and the expression of IL-13 mRNA in the lesional skin both increased, which revealed the Th2-type immune responses induced by DfE and demonstrated the potent allergenic effects of DfE. It is noteworthy that the expression of IL-13 mRNA was higher for the DfE group than that of the DNFB+DfE group in mice dorsal skins. As the Th1 and Th2-type cells come to be counteractive, the Th2-type immune responses in the DNFB+DfE group may be downregulated by the DNFB-related Th1-type immune responses.

In conclusion, the BALB/c mouse model, induced by DNFB and DfE, exhibits typical AD-like skin lesions, scratching behavior, histological changes, and Th2-type immune responses, similar to human AD to a certain extent. We recommend this as a reasonable and easily reproducible method to construct AD-like mouse models. This model would be valuable for studying the pathogenesis of AD and developing novel therapeutic agents for it.

## Data Availability Statement

The original contributions presented in the study are included in the article/supplementary material, further inquiries can be directed to the corresponding author/s.

## Ethics Statement

The animal study was reviewed and approved by Laboratory Animal Welfare and Ethics Committee of Third Military Medical University.

## Author Contributions

SF and WL designed the experiments. SF, WL, and ZZ performed the research, contributed to analysis, and interpretation of data. SF drafted the manuscript. SD, GS, and JZ reviewed the paper. ZS directed the study and reviewed the paper. All authors read and approved the final manuscript.

## Funding

This work was supported by the National Natural Science Foundation of China (Grant No. 82073442).

## Conflict of Interest

The authors declare that the research was conducted in the absence of any commercial or financial relationships that could be construed as a potential conflict of interest.

## Publisher's Note

All claims expressed in this article are solely those of the authors and do not necessarily represent those of their affiliated organizations, or those of the publisher, the editors and the reviewers. Any product that may be evaluated in this article, or claim that may be made by its manufacturer, is not guaranteed or endorsed by the publisher.
